# Integrated Analysis of Gene Co-Expression Network and Prediction Model Indicates Immune-Related Roles of the Identified Biomarkers in Sepsis and Sepsis-Induced Acute Respiratory Distress Syndrome

**DOI:** 10.3389/fimmu.2022.897390

**Published:** 2022-06-30

**Authors:** Tingqian Ming, Mingyou Dong, Xuemin Song, Xingqiao Li, Qian Kong, Qing Fang, Jie Wang, Xiaojing Wu, Zhongyuan Xia

**Affiliations:** ^1^Department of Anesthesiology, Renmin Hospital, Wuhan University, Wuhan, China; ^2^College of Medical Laboratory Science, Youjiang Medical College for Nationalities, Baise, China; ^3^Department of Anesthesiology and Critical Care Medicine, Zhongnan Hospital, Wuhan University, Wuhan, China; ^4^School of Computer, Wuhan University, Wuhan, China; ^5^Department of Otolaryngology-Head and Neck Surgery, Renmin Hospital, Wuhan University, Wuhan, China

**Keywords:** gene co-expression network analysis, sepsis, ARDS, diagnostic biomarker, prediction model, immune cell infiltration

## Abstract

Sepsis is a series of clinical syndromes caused by immunological response to severe infection. As the most important and common complication of sepsis, acute respiratory distress syndrome (ARDS) is associated with poor outcomes and high medical expenses. However, well-described studies of analysis-based researches, especially related bioinformatics analysis on revealing specific targets and underlying molecular mechanisms of sepsis and sepsis-induced ARDS (sepsis/se-ARDS), still remain limited and delayed despite the era of data-driven medicine. In this report, weight gene co-expression network based on data from a public database was constructed to identify the key modules and screen the hub genes. Functional annotation by enrichment analysis of the modular genes also demonstrated the key biological processes and signaling pathway; among which, extensive immune-involved enrichment was remarkably associated with sepsis/se-ARDS. Based on the differential expression analysis, least absolute shrink and selection operator, and multivariable logistic regression analysis of the screened hub genes, *SIGLEC9, TSPO, CKS1B* and *PTTG3P* were identified as the candidate biomarkers for the further analysis. Accordingly, a four-gene-based model for diagnostic prediction assessment was established and then developed by sepsis/se-ARDS risk nomogram, whose efficiency was verified by calibration curves and decision curve analyses. In addition, various machine learning algorithms were also applied to develop extra models based on the four genes. Receiver operating characteristic curve analysis proved the great diagnostic and predictive performance of these models, and the multivariable logistic regression of the model was still found to be the best as further verified again by the internal test, training, and external validation cohorts. During the development of sepsis/se-ARDS, the expressions of the identified biomarkers including *SIGLEC9, TSPO, CKS1B* and *PTTG3P* were all regulated remarkably and generally exhibited notable correlations with the stages of sepsis/se-ARDS. Moreover, the expression levels of these four genes were substantially correlated during sepsis/se-ARDS. Analysis of immune infiltration showed that multiple immune cells, neutrophils and monocytes in particular, might be closely involved in the process of sepsis/se-ARDS. Besides, *SIGLEC9, TSPO, CKS1B* and *PTTG3P* were considerably correlated with the infiltration of various immune cells including neutrophils and monocytes during sepsis/se-ARDS. The discovery of relevant gene co-expression network and immune signatures might provide novel insights into the pathophysiology of sepsis/se-ARDS.

## Introduction

Sepsis has become a medical emergency worldwide with high mortality rates and is usually a result of severe bacterial infection, as well as viral, fungal, or parasitic infection sporadically (1). Most of the severe respiratory conditions were caused by pneumonia, including the currently widespread and life-threatening coronavirus disease-2019 (COVID-19) caused by the severe acute respiratory syndrome coronavirus 2 (2). Acute respiratory distress syndrome (ARDS) is an acute diffuse lung injury developed from multiple clinical conditions including pneumonia, lung contusion, drowning, toxic inhalation, severe systemic infection, severe multiple injuries, shock, high-risk surgery, and pancreatitis (3). Besides, sepsis-induced ARDS (se-ARDS) is often correlated with the high mortality or long-term disability of sepsis (4). However, its complicated clinical features and non-specific molecular characteristics remain an intractable medical problem in achieving the personalized risk evaluation, early prediction, molecular diagnosis, disease recognition, effective prevention, therapy, and prognoses of sepsis and sepsis-induced ARDS (sepsis/se-ARDS) (5).

It has been widely accepted that sepsis is characterized by a severe inflammatory response to infection (6). Increasing evidence has proven that infection-driven systemic inflammatory immunological response and dysfunction play an important part in the development and progression of sepsis/se-ARDS (7–9). Accordingly, the identification of diagnostic biomarkers, the construction of a prediction model, and the illustration of the immunoregulatory pathogenesis could be of major clinical significance for improving the strategies for sepsis/se-ARDS.

In the era of data-driven medicine, large numbers of analysis-based researches, especially bioinformatics analysis, have erupted and penetrated into the field of infection-related diseases, including sepsis/se-ARDS (10, 11). However, most of these studies focused on the primary stages of simple differential expression and functional analysis (12–15). Well-described studies on the regulation of gene expression network, the immune landscape, and the molecular mechanisms and underlying pathogenesis of sepsis/se-ARDS are limited and delayed (15). More in-depth studies, especially analysis with satisfactory and applicable results, are essential and urgently needed in clinical practice.

In the present study, we aimed to explore early genetic regulations based on gene co-expression network, identify novel diagnostic targets, and establish potential risk prediction model for sepsis/se-ARDS by bioinformatics approach. More significantly, this study might help develop potential biological mechanisms and provide more innovative insights into the pathophysiology of sepsis/se-ARDS, which could contribute to clinical prevention, therapy, and prognosis.

## Materials and Methods

### Data Sources and Preprocessing

The RNA-sequencing datasets used in this study were downloaded from the Gene Expression Omnibus (GEO) database of the National Center for Biotechnology Information (16). The GSE32707 dataset included array-based gene expression profiles of whole blood from critically ill patients on the day of admission (day 0) and 7 days later under the platform, GPL10558. Patients from three different groups (34 control patients, 58 sepsis patients and 31 se-ARDS patients) with the uniformly distributed demographic and clinical characteristics of the primary data demonstrated in the original microarray study (17) in GSE32707 were chosen for weighted gene co-expression network analysis (WGCNA), differential expression analysis, model construction and validation, immune infiltration analysis, and the final correlation analysis in this study. In addition, GSE28570 and GSE57065 datasets were also collected for the following independent external validation to verify the diagnostic performance of the sepsis/se-ARDS prediction model. All the criteria of patients identified with sepsis and ARDS were strictly keep to the original researches from the GEO database, and R language was used for raw data preprocessing and the subsequent statistical analysis in this study (18).

### Co-Expression Network Construction

WGCNA was performed by R to construct the co-expression network of genes and the “quantile” function in R were applied to screen the top 25% genes with greatest variance in the GSE32707 dataset (19). After the outlier samples were removed, the cluster dendrogram of the samples was constructed, and then a suitable soft-thresholding power value was determined with the value of R^2^ maximum. During module construction, the similarity matrix was transformed into an adjacency matrix, whereas different adjacencies were calculated and transformed into a consensus topological overlap matrix (TOM). TOM dissimilarity (dissTOM) was computed to construct a network heatmap plot. Highly interconnected genes were separated based on their connectivity and covariance coefficients, and then hierarchically clustered as gene modules, which were identified and assigned with unique color labels. Finally, we estimated module–trait associations by performing consensus module detection under different clinical traits to evaluate specific modules related to sepsis/se-ARDS.

### Identification of Clinically Significant Modules and Hub Genes

Hub genes are highly interconnected with the nodes of the module and are of functional importance for co-expression analysis. The overall expression level of each module represented as module eigengene (ME) was summarized. The correlation between the clinical traits and gene expression of samples was analyzed using R software. The Pearson correlation coefficients between the MEs of each module and each clinical trait were also calculated, where *P*<0.05 indicates a significant correlation between a module and clinical traits in the study. Therefore, these modules were chosen as the key modules.

Gene significance (GS) values were calculated as the Pearson correlation coefficients between the expression levels of every gene and every clinical trait, and module membership (MM) values were also calculated as the Pearson correlation coefficients between the gene expression levels and the ME (19). The genes in the key modules with high GS (>0.3) and high MM (>0.8) were recognized as hub genes.

### Functional and Pathway Enrichment Analyses

The genes in the key module were all subjected to Gene Ontology (GO) and Kyoto Encyclopedia of Genes and Genomes (KEGG) pathway enrichment analyses which were performed *via* the Database for Annotation, Visualization, and Integrated Discovery (DAVID) tools (20). Gene set enrichment analysis (GSEA) was also performed by using GSEA software to identify gseGO function and gseKEGG pathway (21). Biological process (BP) terms were used to represent functional enrichment in GO, “clusterProfiler” package in R was utilized to visualize all the results of DAVID and GSEA, and adjusted *P*<0.05 was regarded statistically significant for enrichment analysis (22).

Gene set variation analysis (GSVA), a novel method of enrichment analysis, was carried out by using “GSVA” package in R (23) to reveal the upregulated or downregulated functional and pathway enrichment terms of a set of modular genes. Their expression profile also played a part in the analysis. The scores for the enriched GO terms and KEGG pathways were calculated based on the expression levels of the modular genes. Afterward, the GSVA scores to quantify functional and pathway enrichments between sepsis/se-ARDS and healthy controls were compared, evaluated, and visualized in the “limma” package in R with adjusted *P*<0.05.

### Verification of the Differential Expression of Hub Genes

The expression profiles of hub genes in the GSE32707 dataset were extracted and constructed by TBtools software (Toolbox for Biologist v1.09854) in the same samples as WGCNA (24). Differential expression analysis between healthy and sepsis/se-ARDS samples was conducted using the “limma” package in R to verify the differential expression of these hub genes (25). All the hub genes were detected and are shown in the differentially expressed volcano diagram with their gene symbols presented in the figure. In this study, log_2_(fold change)>0.5 and −log_10_(adj. *P*)>1.122 (adj. *P*<0.05) were the screening thresholds.

### LASSO Regression and Multivariable Logistic Regression Analysis

The least absolute shrinkage and selection operator (LASSO) algorithm was executed by the “glmnet” package in R to reduce the high dimensions of data; the hub genes obtained from WGCNA were used to screen the potential biomarkers; and the variables with nonzero coefficients were selected as the optimal genes and entered into the subsequent multivariable logistic regression analysis in R (26). A prediction model that incorporates the genes screened from the LASSO regression was constructed using *P*-value and odds ratio (OR) with 95% confidence interval (CI) as features to identify the diagnostic biomarker candidates.

### Nomogram Model Construction and Decision Curve Analysis

The final variables from the multivariable logistic regression analysis were identified as the potential predictors and diagnostic biomarkers associated with sepsis/se-ARDS. Afterward, a risk prediction nomogram model was conducted and assessed in R by calibration curves and Harrell’s *C*-index calculation (27). Decision curve analysis was also performed in R to determine the clinical usefulness of the nomogram by quantifying the net benefits at different threshold probabilities in the sepsis/se-ARDS cohorts.

### Receiver Operating Characteristic (ROC) Curve Analysis

The samples in the GSE32707 dataset were separated into sepsis and se-ARDS groups, as well as into training set (75%) and test set (25%), in order to verify and validate the screened gene biomarkers based on the diagnostic model. Two independent datasets (GSE28570 and GSE57065) were employed as external validation sets. ROC curve analyses, including the specificity, sensitivity, likelihood ratios, positive predictive values, negative predictive values, and area under ROC curve (AUC), were conducted in the training, test, and validation sets (28) and then calculated and drawn by the “pROC” package in R to evaluate the predictive efficiency of the prediction models established in the study (29) and assess the diagnostic value of the screened genetic biomarkers used in the construction of the models.

### Machine Learning Algorithms

After ROC analysis was conducted for model construction, five different popular classification algorithms, namely, Support Vector Machines (SVMs), Random Forest (RF), Naïve Bayes (NB), Neural Network (NN), and Flexible Discriminant Analysis (FDA), were used to develop and conduct machine learning-based models (30) and evaluate the model’s performance in predicting the occurrence of sepsis/se-ARDS. These processes were performed using the “caret” package in R.

### Gene Expression Regulation and Correlation Analysis

The regulation of gene expression during the development of sepsis/se-ARDS was investigated. The expression profile of the GSE32707 dataset was utilized to analyze the expression variation trends of the gene markers among the healthy control group, sepsis groups on days 0 (admission day) and 7, and se-ARDS groups on days 0 and 7, where the expression analysis was additionally performed in the external cohorts of GSE28750 and GSE57065 datasets to validate the regulations of specific genes. Then, multiple comparisons between groups were executed separately for the comprehensive analysis of marker gene expression during sepsis/se-ARDS development.

Moreover, Pearson correlation analysis was performed to further analyze the correlation between the disease status of samples and the expression of the identified diagnostic biomarkers, as well as the correlation of different biomarkers’ expression during sepsis/se-ARDS development.

*P*<0.05 was considered statistically significant. All the results were visualized by the “ggplot2” package in R.

### Immune Infiltration and Correlation Analysis With Diagnostic Biomarkers During Sepsis/Se-ARDS Development

We performed immune infiltration analysis by CIBERSORT to evaluate the profiles of 22 kinds of infiltrating immune cells based on all the samples from healthy controls and sepsis/se-ARDS groups in the GSE32707 dataset. The matrix data of gene expression were uploaded, and then CIBERSORT was used to obtain the immune cell infiltration matrix for the following analysis, and visualization was performed using the “ggplot2” package in R (31). Bar-plot, heatmap, and violin diagram were drawn to compare and evaluate the differences in immune infiltration among the samples. Moreover, the correlation of the 22 types of infiltrating immune cells among the samples was performed and visualized by a correlation heatmap in R.

Pearson correlation analysis was performed and visualized by the “ggplot2” package in R to analyze the correlation between the expression of the identified diagnostic biomarkers and infiltrating immune cells during sepsis/se-ARDS development.

## Results

### Weighted Gene Co-Expression Networks and Module Construction Analysis

**Figure 1:**
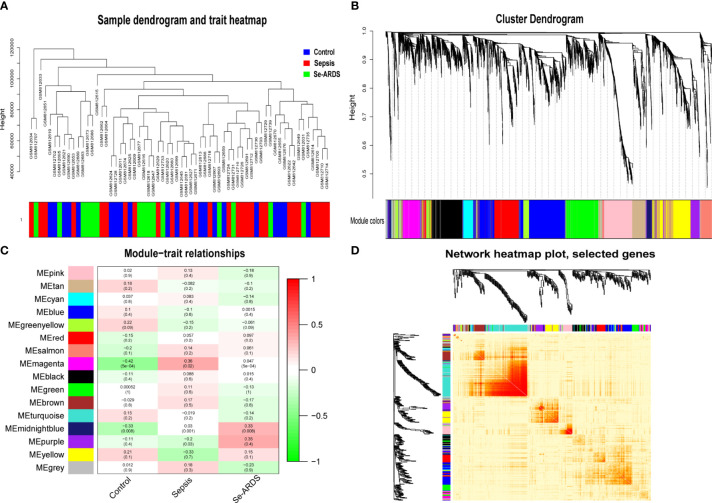
Co-expression network and module–trait relations of sepsis/se-ARDS. **(A)** Sample dendrogram and trait heatmap of sepsis/se-ARDS. **(B)** Cluster dendrogram of all genes based on key modules. **(C)** Co-expression network heatmap based on dissTOM. **(D)** Relationships of modules with clinical traits.

WGCNA was carried out to identify the co-expression network of sepsis/se-ARDS-associated genes in the GSE32707 dataset. A total of 31311 genes expressed in 123 samples whose clinical characteristics could be obtained from the original study (17) were calculated by applying “quantile” function in R, and accordingly, 7828 genes in the top 25% greatest variance genes were screened to identify co-expression patterns. The outgroup samples were removed (**Supplementary Figure S1A**), and the dendrogram and trait heatmap of the remaining 82 samples were displayed in **Figure 1A**. Based on the criteria for combining the scale-free topology fit index of R^2^ and effective mean connectivity (**Supplementary Figures S1B, C**), we selected 9 as the optimal soft-thresholding power for later analysis. The co-expression networks were then constructed. The cluster dendrogram and network heatmap of all the genes were presented in **Figures 1B, C**, respectively. Sixteen consensus gene co-expression modules were obtained, and their interactions were presented by hierarchical clustering analysis as the module eigengene dendrogram and eigengene network heatmap (**Supplementary Figure S1D**).

### Identification of Key Consensus Modules and Hub Genes Associated With Sepsis/Se-ARDS

All of the consensus gene co-expression modules were analyzed to identify the crosstalk between the consensus modules and clinical traits. As shown in **Figure 1D**, the module–trait relationships indicated that the magenta and midnight blue modules were remarkably correlated with the clinical traits of the healthy control, sepsis, and se-ARDS groups.

**Figure 2:**
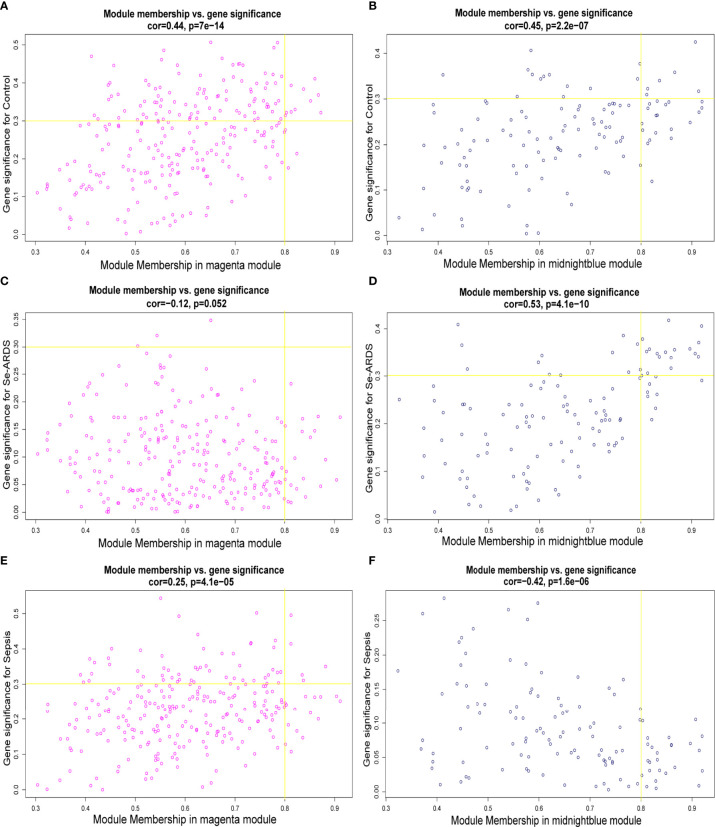
Identification of hub genes associated with sepsis/se-ARDS. Correlation between module membership (MM) and gene significance (GS) for the **(A, B)** control, **(C, D)** sepsis, and **(E, F)** se-ARDS groups in the key magenta (left) and midnight blue (right) modules.

Therefore, the magenta and midnight blue modules were identified as the key related consensus gene modules which were separated based on the connectivity and covariance coefficients of highly interconnected genes for the final hierarchical clusters, and the involved 384 modular genes obtained from the above key magenta and midnight blue modules, which were remarkably correlated with the clinical traits in this study, were listed in **Supplementary Table S1** and then used for the screening of hub genes associated with sepsis/se-ARDS. The relationship between GS and the intramodular connectivity of the magenta and midnight blue modules in different groups was proven to be substantial as indicated in **Supplementary Figures S2A–C**. fThe correlation between GS and MM was explored, and the results shown in **Figures 2A–F** demonstrated that the magenta and midnight blue modules were considerably associated with all the clinical traits of the control, sepsis, and se-ARDS groups. Accordingly, the heatmaps of the involved genes in the magenta and midnight blue modules among samples were displayed in **Supplementary Figures S3A, B** and the top36 genes in the magenta and midnight blue modules were screened and identified as hub genes using the criteria: MM>0.8 and GS>0.3 (**Supplementary Table S2**).

### Functional and Pathway Enrichment Analyses of Modular Genes Involved in Sepsis/Se-ARDS

The enrichment of the 384 modular genes with their expression levels in samples was studied via a comprehensive analysis based on DAVID, GSEA, and GSVA (**Supplementary Figures S4A–D**).

**Figure 3:**
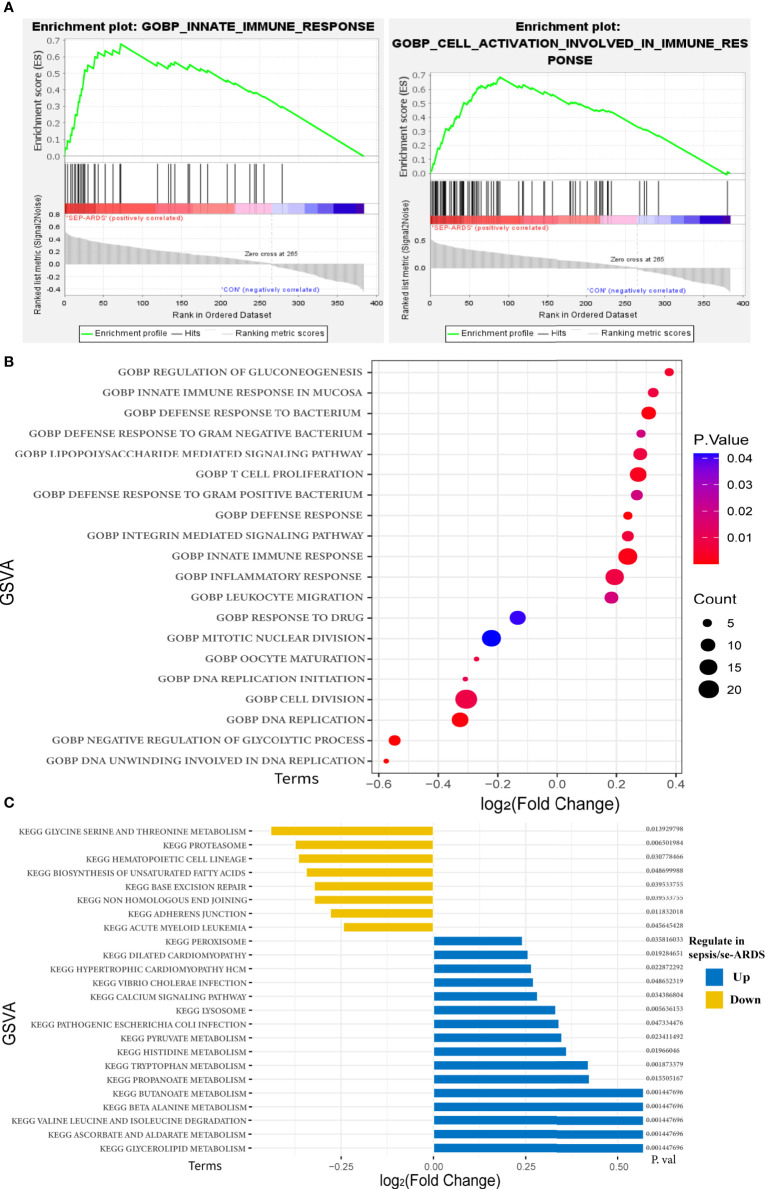
Functional and pathway enrichment analyses of the key modules of sepsis/se-ARDS. **(A)** GSEA of innate immune response (left) and cell activation involved in immune response (right). **(B)** GSVA-based analysis of biological function enrichment by bubble plot. **(C)** GSVA-based analysis of KEGG pathway enrichment by bar-plot.

Notably, the results of GO enrichment performed by DAVID showed that innate immune response in the mucosa, defense response to bacterium, lipopolysaccharide-mediated signaling pathway, and inflammatory response were remarkably upregulated in sepsis/se-ARDS compared with the control, whereas response to drug, mitotic nuclear division, cell division, and DNA replication were substantially downregulated. KEGG pathway analysis showed that lysosome, peroxisome, calcium signaling pathway, and glycerolipid metabolism were considerably upregulated in sepsis/se-ARDS compared with the control. Conversely, hematopoietic cell lineage, proteasome, glycine serine, and threonine metabolism were markedly downregulated. The calculation of BP terms in GSEA also revealed and confirmed that inflammation and immunity-related gene sets, such as innate immune response and cell activation involved in immune response, were remarkably enriched in sepsis/se-ARDS (**Figure 3A**). In summary, the enrichment scores of common terms were qualified by GSVA and visualized in R as exhibited in **Figures 3B, C**.

In addition, in order to acquire more details for understanding the picture of the hub genes, the above enrichment analyses were also performed in the 36 hub genes identified previously. Similarly, the GSVA scores of GO enrichments and KEGG pathways shown in **Supplementary Figures S5A, B** also demonstrated that quite a few terms of GO annotations and KEGG pathways were coincidentally enriched such as immune response, inflammation and metabolism, which validated the consistency of the enriched functional terms and pathways in this study.

### Construction of Diagnostic Prediction Model for Sepsis/Se-ARDS

**Figure 4:**
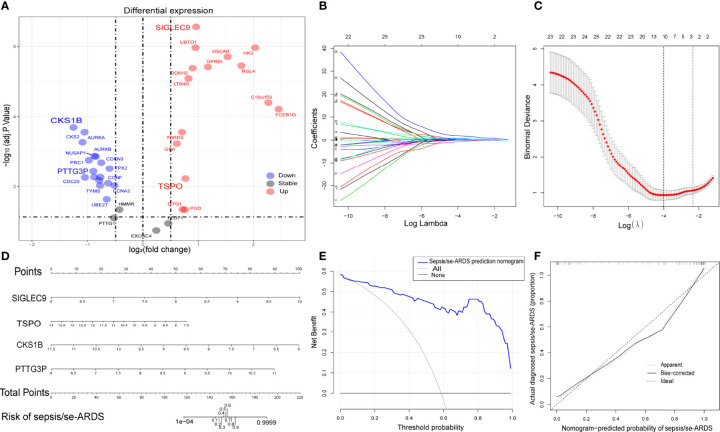
Construction of diagnostic prediction model for sepsis/se-ARDS. **(A)** Thirty-six hub genes differentially expressed between sepsis/se-ARDS samples and controls. **(B)** Different coefficients values and **(C)** binomial deviance values within the range of lambda. **(D)** Nomogram model for sepsis/se-ARDS prediction based on the identified biomarkers, *SIGLEC9*, *TSPO*, *CKS1B*, and *PTTG3P*. **(E)** Decision curve analysis and **(F)** calibration curve analysis of the sepsis/se-ARDS risk nomogram model.

The differential expression of the 36 hub genes obtained from WGCNA was detected and identified by differential expression analysis to verify their potential diagnostic prediction value. **Figure 4A** presented all the hub genes with their gene symbols in a volcano diagram. Thirty-two hub genes were differentially expressed and entered into the subsequent model construction.

Based on the LASSO algorithm, the 32 hub genes were reduced into 10 potential predictors (lambda. min=0.01824334, **Figures 4B, C**). These 10 variables with nonzero coefficients were subjected to the following multivariable logistic regression analysis. As indicated in **Table 1**, four genes, namely, *SIGLEC9*, *TSPO*, *CKS1B*, and *PTTG3P*, stood out of the 10 genes and were recognized as diagnostic biomarker candidates for sepsis/se-ARDS. A second round of multivariable logistic regression analysis was performed to eliminate unnecessary interference of the insubstantial variables and then to construct a more practicable diagnostic prediction model focused on these four genes (**Table 2**).

**Table 1:** Results of multiple logistic regression analysis based on 10 hub genes screened from LASSO regression.

Variable	Regression Coefficient	Std. Error	OR (95%CI)	z-value	*p*-Value
(Intercept)	-28.492	11.49144	4.228e-13 (1.620e-24, 4.448e-04)	-2.479	0.0132*
*DDAH2*	-0.20547	1.95753	0.814 (1.781e-02, 3.997e+01)	-0.105	0.9164
*OSCAR*	-0.03748	0.94491	0.963 (1.416871e-01, 6.491463e+00)	-0.04	0.9684
***SIGLEC9***	5.43723	2.24103	229.8053 (4.952e+00, 3.675e+04)	2.426	0.0153*
*GPR84*	0.65854	0.94666	1.932 (3.635e-01, 1.442e+01)	0.696	0.4866
*LTB4R*	0.9101	1.16046	2.485 (2.383e-01, 2.502e+01)	0.784	0.4329
***TSPO***	-2.28203	0.89929	0.102 (9.725e-03, 4.604e-01)	-2.538	0.0112*
***CKS1B***	-3.68616	1.61789	0.0251 (6.925e-04, 4.371e-01)	-2.278	0.0227*
*UBTD1*	0.2199	1.28837	1.246 (1.069e-01, 1.745e+01)	0.171	0.8645
***PTTG3P***	3.86828	1.53974	47.860 (3.230e+00, 1.556e+03)	2.512	0.0120*
*TDRD9*	0.12884	1.02718	1.138 (1.616e-01, 9.624e+00)	0.125	0.9002

*P<0.05. Diagnostic biomarker candidates are indicated in bold.

LASSO, least absolute shrink and selection operator; OR, odds ratio; CI, confidence interval; DDAH2, dimethylarginine dimethyl amino hydrolase 2; OSCAR, osteoclast associated Ig-like receptor; SIGLEC9, sialic acid-binding Ig-like lectin 9; GPR84, G protein-coupled receptor 84; LTB4R, leukotriene B4 receptor; TSPO, translocator protein; CKS1B, cyclin-dependent kinase regulatory subunit 1B; UBTD1, ubiquitin domain containing 1; PTTG3P, putative pituitary tumor-transforming gene 3 protein; TDRD9, tudor domain containing 9.

**Table 2:** Results of multiple logistic regression analysis based on the four key genes.

Variable	Regression Coefficient	Std. Error	OR (95%CI)	z-value	*p*-Value
(Intercept)	-21.7946	7.9037	3.425e-10 (7.470e-18, 5.800e-04)	-2.758	0.00582**
***SIGLEC9***	6.2852	1.5547	5.366e+02 (4.008e+01, 1.997e+04)	4.043	5.28e-05***
***TSPO***	-2.0966	0.7011	1.229e-01 (2.386e-02, 3.868e-01)	-2.99	0.00279**
***CKS1B***	-4.5461	1.3966	1.061e-02 (4.531e-04, 1.194e-01)	-3.255	0.00113**
***PTTG3P***	4.5038	1.3778	9.036e+01 (8.279e+00, 2.046e+03)	3.269	0.00108**

**P<0.01; ***P<0.001. Identified diagnostic biomarkers are indicated in bold.

OR, odds ratio; CI, confidence interval; SIGLEC9, sialic acid-binding Ig-like lectin 9; TSPO, translocator protein; CKS1B, cyclin-dependent kinase regulatory subunit 1B; PTTG3P, putative pituitary tumor-transforming gene 3 protein.

Afterwards, a sepsis/se-ARDS risk nomogram model that involves *SIGLEC9*, *TSPO*, *CKS1B*, and *PTTG3P* as the independent predictors was constructed (**Figure 4C**). The calibration curve of the nomogram for sepsis/se-ARDS risk prediction demonstrated great agreement with the actually diagnosed probability of sepsis/se-ARDS (**Figure 4D**). The *C*-index of the prediction nomogram was 0.950 (95% CI=0.928–0.972). Moreover, the results of the decision curve and calibration curve analyses (**Figures 4E, F**) for the sepsis/se-ARDS risk nomogram exhibited apparent performance with excellent discrimination and prediction capability.

Furthermore, additional four-gene models based on different machine learning methods were established for the validation analysis of the identified biomarker signatures. It was indicated in **Table 3** that based on their respective optimum parameters, all the five machine learning models exhibited great classification performance in predicting sepsis/se-ARDS in regard to ROC analysis.

**Table 3:** Diagnostic performances of the four-gene models based on machine learning algorithms.

Model	Optimum Parameter	Sensitivity	Specificity	ROC
SVM	sigma=0.007904905, C=0.25	0.3846667	0.8799167	0.7833028*
RF	mtry=43	0.7056000	0.8731167	0.8977694**
NB	lapplace=0, usekernel=TRUE, adjust=1	1	0.005625	0.8207542**
NN	Size=5, decay=0.1	0.3486833	0.9781583	0.7925806*
FDA	degree=1, nprune=2	0.7183500	0.7919083	0.8511097**

*0.70≤ROC<0.80, good; **0.80≤ROC<0.90, great.

ROC, receiver operating characteristic; SVMs, Support Vector Machines; RF, Random Forest; NB, Naïve Bayes; NN, Neural Network; FDA, Flexible Discriminant Analysis.

### Verification and Validation of Diagnostic Biomarkers and Prediction Models for Sepsis/Se-ARDS

**Figure 5:**
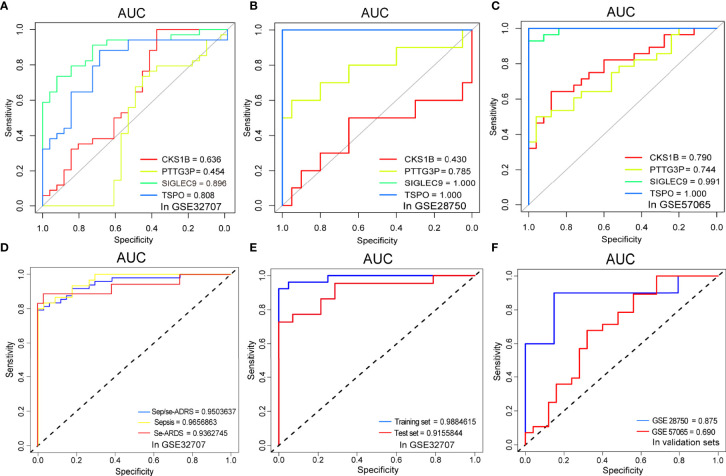
Comparisons of ROC curves and AUC performances. The ROC curve analysis of sepsis/se-ARDS diagnostic efficacy of *SIGLEC9*, *TSPO*, *CKS1B*, and *PTTG3P* in **(A)** GSE32707, **(B)** GSE28750 and **(C)** GSE57065. **(D)** The ROC curve analysis of sepsis/se-ARDS prediction efficacy of the four-gene model in sepsis, se-ARDS or sepsis/se-ARDS cohorts(left) and **(E)** in training or test cohorts (right) of GSE32707. **(F)** The ROC curve analysis of sepsis/se-ARDS prediction efficacy of the four-gene models in validation cohorts of GSE28750 and GSE57065.

ROC curve analyses verified and validated the prediction efficacy of the diagnostic biomarker candidates, *SIGLEC9*, *TSPO*, *CKS1B*, and *PTTG3P*, as well as the prediction models based on the foregoing analysis. The AUCs of the respective diagnostic values of the four genes for sepsis/se-ARDS in GSE32707, GSE28570 and GSE57065 were detected. And as displayed in **Figures 5A–C**, most of these four genes especially *SIGLEC9* and *TSPO* exhibited great diagnostic values for sepsis/se-ARDS. The prediction performances of the models based on the four biomarkers were evaluated in different cohorts. Interestingly, all these models exhibited substantial prediction efficiency in sepsis and se-ARDS cohorts, as well as in the training, test, or validation cohorts (**Figures 5D–F**).

### Analysis of Marker Gene Expressions During Sepsis/Se-ARDS Development

**Figure 6:**
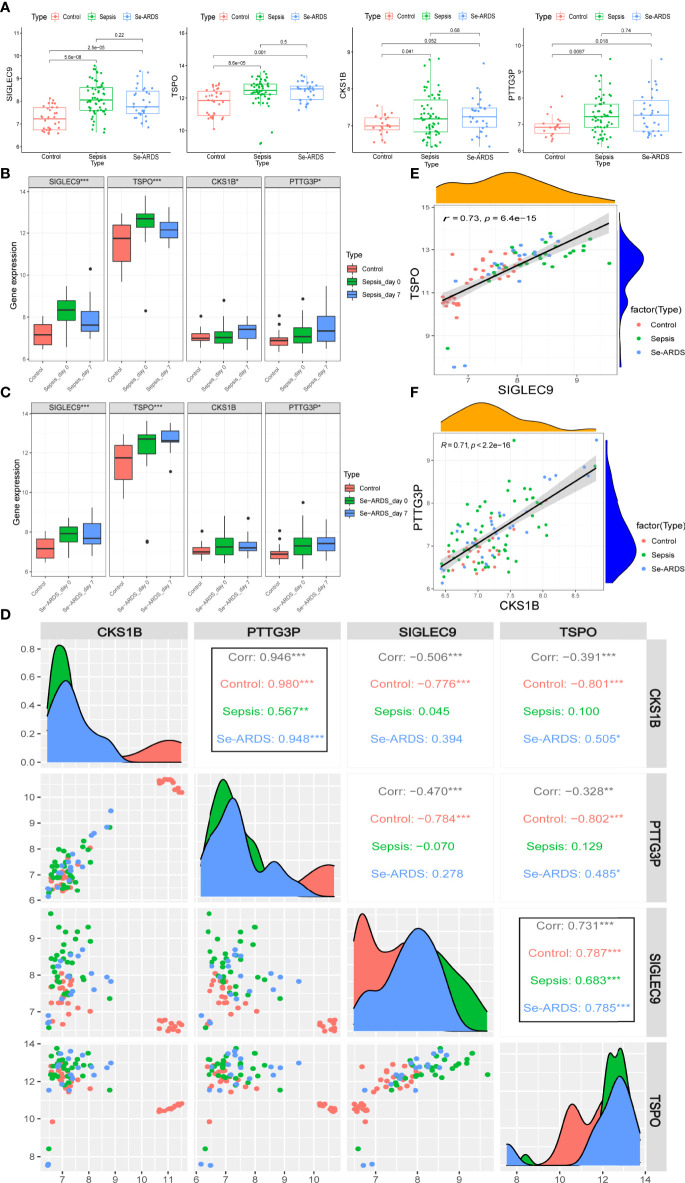
Expression of marker genes during sepsis/se-ARDS development. **(A)** Expression regulation of *SIGLEC9*, *TSPO*, *CKS1B*, and *PTTG3P* during the course of sepsis/se-ARDS. **(B)** Expression variation trends of *SIGLEC9*, *TSPO*, *CKS1B*, and *PTTG3P* among the control, sepsis day 0 and 7 groups. **(C)** Expression variation trends of *CKS1B*, *PTTG3P*, *SIGLEC9*, and *TSPO* among the control, se-ARDS day 0 and 7 groups. **(D)** Correlation analysis of the four biomarkers during sepsis/se-ARDS development. Correlation analysis between **(E)** *SIGLEC9* and *TSPO* and between **(F)** *CKS1B* and *PTTG3P*. *P < 0.05; **P < 0.01; ***P < 0.001.

The regulations of marker gene expressions during the course of sepsis/se-ARDS were detected in samples of GSE32707 and were presented in **Figure 6A**. All of these four identified biomarkers, *SIGLEC9*, *TSPO*, *CKS1B*, and *PTTG3P*, were remarkably upregulated in the sepsis and se-ARDS group compared with the control group (*P*<0.05, except for *CKS1B* in se-ARDS group), whereas no remarkable changes were shown in the se-ARDS group compared with the sepsis groups (*P*>0.05). Moreover, the expression analysis of these four genes in sepsis condition was also additionally performed in the external cohorts of GSE28750 and GSE57065 to validate their consistency in regulations of gene expressions. As demonstrated in **Supplementary Figures S6A-D**, *SIGLEC9*, *TSPO*, *CKS1B*, and *PTTG3P* all exhibited remarkable changes of upregulations in the sepsis group compared with control group in both datasets with *P*<0.05 (except for *CKS1B* in GSE28750), which proved the great diagnostic and predictive performance of these four biomarkers.

The expression variation trends of the gene markers were analyzed among different groups of healthy control, patients on days 0 (admission day) and 7 of sepsis/se-ARDS. As displayed in **Figures 6B**, both *SIGLEC9* and *TSPO* were significantly upregulated on sepsis day 0 and then descended a bit more on sepsis day 7 (***P<0.001) during sepsis development, while both *CKS1B* and *PTTG3P* appeared to be steadily upregulated on sepsis day 0 and day 7 (*P<0.05). Similar alterations were observed in the expressions of *SIGLEC9*, *TSPO* and *PTTG3P* during se-ARDS development, whereas *CKS1B* was not regulated significantly (**Figures 6C**).

Further correlation analysis based on *SIGLEC9*, *TSPO*, *CKS1B*, and *PTTG3P* was carried out to identify the potential interactions and effects of these diagnostic biomarkers during sepsis/se-ARDS development. As displayed in **Figure 6D**, the expressions of four genes were remarkably related to each other, and it is apparent that the highly positive correlations between *SIGLEC9* and *TSPO* and between *CKS1B* and *PTTG3P* were spectacularly remarkable at all the stages of sepsis/se-ARDS progression (**Figures 6E, F**).

### Landscape of Immune Cell Infiltration and Its Correlation Analysis With Diagnostic Biomarkers During Sepsis/Se-ARDS Development

**Figure 7:**
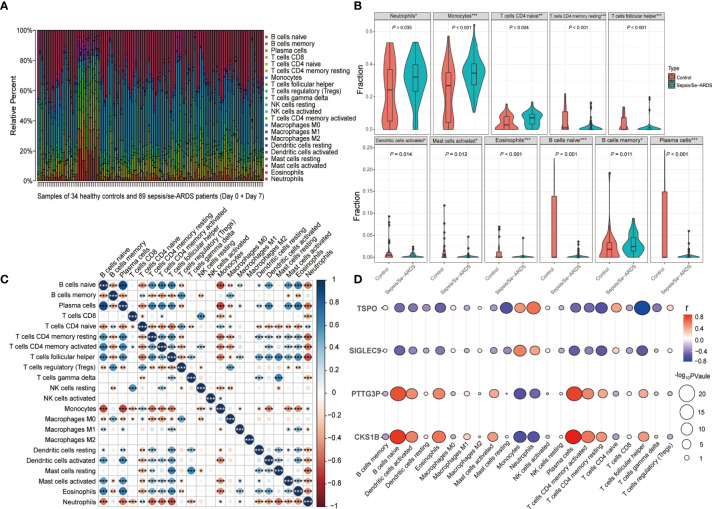
Analysis of immune cell infiltration during sepsis/se-ARDS development. **(A)** Hierarchical clustering heatmap of the subpopulations of 22 types of infiltrating immune cells among all samples. **(B)** Violin plot of the significantly dysregulated immune cells in sepsis/se-ARDS. **(C)** Correlation analysis of the 22 types of infiltrating immune cells. **(D)** Correlation analysis of the four biomarkers with the levels of immune cell infiltration. *P < 0.05; **P < 0.01; ***P < 0.001.

The immune infiltration analysis performed by CIBERSORT summarized the infiltrating levels of 22 types of immune cells among all the samples from healthy controls and sepsis/se-ARDS patients in GSE32707 dataset, and the results were presented as the relative infiltrating proportions (**Supplementary Figure S7A**). The hierarchical clustering heatmap of subpopulations was shown in **Figure 7A**. The comparisons of every immune cell infiltration between groups were analyzed in GSE32707 and presented with the significantly dysregulated results of infiltrating immune cells indicated in **Figure 7B** and the remaining results with no remarkable difference were indicated in **Supplementary Figure S7B**, which have been verified again by the same analysis in external validation cohorts of GSE28570 and GSE57065 as exhibited in **Supplementary Figures S7C, D**. Compared with the control group, multiple types of immune cells especially higher levels of neutrophils (*P*=0.035 in GSE32707, 0.007 in GSE28750 and <0.001 in GSE57065) and monocytes (*P*<0.001 in GSE32707, 0.031 in GSE28750 and 0.028 in GSE57065) were repeatedly differentially infiltrated in the samples from the sepsis/se-ARDS groups (**Figure 7B** and **Supplementary Figure S4B**). In addition, as displayed in **Figure 7C**, the detailed correlation analysis of the 22 types of immune cells demonstrated that almost all infiltrating immune cells except for macrophages M2 were significantly correlated with specific types of immune cells partly, where both neutrophils and monocytes exhibited strong correlations with substantial immune cells (*P<0.05; **P<0.01; ***P<0.001).

Moreover, the levels of immune cell infiltration in the progress of sepsis/se-ARDS promoted their further correlation analysis based on *SIGLEC9*, *TSPO*, *CKS1B*, and *PTTG3P*, the diagnostic biomarkers identified in the study. It was revealed in **Figure 7D** that these four genes, all demonstrated good correlations with various infiltrating immune cells. As the identified immune cell types, neutrophils and monocytes in particular, might suggest a remarkable dysregulation during the pathological process, and their correlations with these four genes were analyzed accordingly. In addition, both *SIGLEC9* and *TSPO* showed significant positive correlations with the infiltration levels of both neutrophils and monocytes (*SIGLEC9*: *r*=0.45, *P*=2.3e−07 for neutrophils and *r*=0. 58, *P*=1.2e−12 for monocytes; *TSPO*: *r*=0.64, *P*=1.8e−15 for neutrophils and *r*=0.5, *P*=3.8e−09 for monocytes) as displayed in **Supplementary Figures S8A-D**, while both *CKS1B* and *PTTG3P* showed significant negative correlations with the infiltration levels of both neutrophils and monocytes (*CKS1B*: *r*=−0.55, *P*=2.8e−11 for neutrophils and *r*=−0.59, *P*=5.2e−13 for monocytes; *PTTG3P*: *r*=−0.46, *P*=7.2e−08 for neutrophils and *r*=−0.56, *P*=1e−11 for monocytes) as displayed in **Supplementary Figures S8E–H**.

Together, these results indicated that these four genes were closely related to each other, and might play important roles in the dysregulated infiltration of specific immune cells during sepsis/se-ARDS development.

## Discussion

Sepsis has emerged as a life-threatening condition, and ARDS induced by sepsis has added massive destructions and posed a great threat worldwide for the unavailability of efficient risk prediction, accurate genetic diagnosis, and molecular intervention; thus, se-ARDS gave rise to poor outcome and prognosis and limited effective prevention and therapy (5). The early prediction, recognition, and diagnosis of sepsis/se-ARDS are critical for appropriate treatment, while bioinformatics analysis provides an ideal method to screen large-scale gene expression data to explore the underlying mechanism of sepsis/se-ARDS (10).

WGCNA has functioned a lot on developing correlation patterns between phenotype and genomics, and the key modules and hub genes from the co-expression network has great importance in bioinformatics (32–34). However, these bioinformatic studies are rarely used in sepsis/se-ARDS (35). LASSO analysis, multivariable logistic regression method, ROC analysis and machine learning algorithms have been proven to act well on the bioinformatics of various diseases (36, 37). Accordingly, we identified *SIGLEC9*, *TSPO*, *CKS1B*, and *PTTG3P* as the diagnostic biomarkers and then established and validated four-gene-based models for the disease prediction. The fantastic performances of the algorithmic models especially the multivariable logistic regression as well as the significant dysregulation of these diagnostic biomarkers proved their remarkable theoretically instructive importance and application value for clinical practice.

Immunoregulation plays an essential role in the pathogenesis of sepsis/se-ARDS (7, 38). The functional enrichment analysis also suggested that immune response was considerably dysregulated in the study, but the detailed landscape of immune infiltration remains unclear and needs to be explored by immune cell infiltration analysis, despite the wide application of CIBERSORT for cancer and immune disease in recent years (39). The present study found that multiple infiltrating immune cells including neutrophils and monocytes in particular might participate in the dysregulated immune response during the progress of sepsis/se-ARDS. Importantly, the correlations of the four identified genes and the above immune infiltration cells with the development of sepsis/se-ARDS were analyzed. Surprisingly, *SIGLEC9*, *TSPO*, *CKS1B*, and *PTTG3P* all exhibited remarkable correlations with neutrophils and monocytes. This finding further consolidates the potential roles of the biomarkers and provide novel insights into the immunotherapeutic targets.

It was revealed in our study that the four identified biomarkers, exhibited substantial differentiation and significant correlations with each other and immune cell infiltrations during sepsis/se-ARDS development. Sialic acid-binding Ig-like lectin 9 (*SIGLEC9*) is a member of Siglecs that are only expressed in immune cells and regulate immune balance in inflammatory diseases (40). It has been revealed that Siglec-9 helps endocytosis of toll-like receptor 4, regulates macrophages polarization, and inhibits the function of neutrophils by mediating death signals in neutrophils during infection in the pathogenesis of sepsis (40, 41). Previous study has proved that septic shock patients exhibit different ex vivo death responses of blood neutrophils after Siglec-9 ligation early in shock (41). A human anti-Siglec-9 Fab fragment, named hS9-Fab03, has been developed and investigated its efficient immune activity for blocking LPS-induced pro-inflammatory cytokines production activity in human macrophages (42) and a recent study reported that synthetic Siglec-9 agonists inhibit neutrophil activation associated with COVID-19 (43). Moreover, *SIGLEC9* has been reported to function a lot on antivirus and antitumor progression by mediating inhibitory immunoregulation as well as immune surveillance (44, 45). Translocator protein (*TSPO*) is a mitochondrial protein implicated in steroidogenesis and inflammatory responses such as cytokine release and oxidative stress (46). It is upregulated in immune cells and has attracted great interest as a biomarker of the neuroinflammatory response. Recent studies have shown that the *TSPO* mRNA and protein were upregulated in the inflammatory brain of the murine endotoxemia model and identified the possible role of *TSPO* in sepsis-associated encephalopathy (SAE) (47). Cyclin-dependent kinase regulatory subunit 1B (*CKS1B*) is crucial in cell cycle regulation and closely related to tumor initiation, maintenance, and progression, thus significantly associated with the prognosis of cancers (48, 49). Putative pituitary tumor-transforming gene 3 protein (*PTTG3P*) was found to be regulated expressed in several tumors, which proved to play a role in interleukin (IL)-1β, IL-8 production and B cell growth and a series of cell functions such as cell growth, migration, invasion and apoptosis (50, 51). In spite of the proven effects of *SIGLEC9* and *TSPO* on sepsis and sepsis-related pathological mechanisms as well as the potent underlying roles of these four genes in immune and inflammatory responses, none of these genes have been studied or explored in se-ARDS at a clinical level. Considering their great performances of diagnostic value and immune correlation in the present study, the potential roles of these identified biomarkers in sepsis/se-ARDS and their underlying mechanisms deserve to be elucidated in the near future.

In regard to sepsis-related bioinformatics studies, the gene expression of the whole blood was obtained and analyzed for potential gene expression dysregulation and novel genetic biomarkers, except for traditional methods, such as clinical biochemical indexes, known protein biomarkers, and inflammatory or immune mediators (52). The use of single biomarkers, little database application, and simple superficial analysis methods led to the tardiness and limitations of the advances in bioinformatics studies on sepsis especially se-ARDS (13). Nowadays, there existed few well-described bioinformatic studies especially on the regulation of gene expression network, the immune landscape, and the molecular mechanisms and underlying pathogenesis of sepsis/se-ARDS (15). The current study applied overall data and advanced means, combined multiple biomarkers, established disease models and explored the in-depth mechanism of sepsis/se-ARDS. By the identification of diagnostic biomarkers, the construction of prediction models, and the illustration of the immunoregulatory pathogenesis, our study was the first to achieve the personalized risk evaluation, early prediction, molecular diagnosis, disease recognition, effective prevention, therapy, and prognoses of sepsis/se-ARDS, which might provide novel insight and certain significance for improving the strategies for future clinical practice.

Regrettably, the study still has some limitations, such as the lack of available clinical data which contained the pre-conditions of samples that could affect the immune response in the analysis and various aspects of novel deep learning or artificial intelligence algorithms (53), biomedical data sciences, precision medicine, and translational bioinformatics approaches. How this impacts the advancement of knowledge in septic patients, how to medically prove the effectiveness and necessity of this prediction model and how to practice the significance of clinical transformation would need more investigations and efforts. More importantly, solid and diverse evidences in demand, including relevant original wet experiments of sepsis/se-ARDS, especially studies on immune-related mechanisms and by specific gene knock-out *in vivo* and *in vitro*, are required to generate a more comprehensive understanding of sepsis/se-ARDS in future medical practice.

## Conclusion

In summary, the present study identified *SIGLEC9*, *TSPO*, *CKS1B*, and *PTTG3P* as the genetic biomarkers and established a four-gene-based model for the effective diagnosis and risk prediction of sepsis/se-ARDS based on gene co-expression network. Immune dysregulation mediated by these four biomarkers might be associated with sepsis/se-ARDS development. The study would provide novel insights into the potential molecular targets to combat sepsis and promote a more comprehensive understanding of the underlying immune mechanism involved in the pathogenesis of sepsis/se-ARDS.

## Data Availability Statement

The datasets presented in this study can be found in online repositories. The names of the repository/repositories and accession number(s) can be found in the article/**Supplementary Material**.

## Ethics Statement

Written informed consent was obtained from the individual(s) for the publication of any potentially identifiable images or data included in this article.

## Author Contributions

TM, XW, and ZX conceived and designed the idea, TM performed the experiments and drafted the manuscript; TM and MD interpreted the results and machine learning method; XS and QF carried out data collection and curation; XL, QK and JW performed some interpretation of the results. All authors read and approved the final version of the manuscript.

## Funding

The present study was supported by grants from the National Natural Science Foundation of China (NO. 81970722, 81901952 and 82172144).

## Conflict of Interest

The authors declare that the research was conducted in the absence of any commercial or financial relationships that could be construed as a potential conflict of interest.

## Publisher’s Note

All claims expressed in this article are solely those of the authors and do not necessarily represent those of their affiliated organizations, or those of the publisher, the editors and the reviewers. Any product that may be evaluated in this article, or claim that may be made by its manufacturer, is not guaranteed or endorsed by the publisher.

## Abbreviations

ARDS, acute respiratory distress syndrome; sepsis/se-ARDS, sepsis and sepsis-induced acute respiratory distress syndrome; LASSO, least absolute shrink and selection operator; ROC, receiver operating characteristic; COVID-19, coronavirus disease-2019; se-ARDS, sepsis-induced ARDS; NCBI, National Center for Biotechnology Information; GEO, Gene Expression Omnibus; WGCNA, weighted gene co-expression network analysis; TOM, topological overlap matrix; dissTOM, dissimilarity of topological overlap matrix; ME, module eigengene; GS, gene significance; MM, module membership; GO, Gene Ontology; KEGG, Kyoto Encyclopedia of Genes and Genomes; DAVID, Database for Annotation, Visualization, and Integrated Discovery; GSEA, gene set enrichment analysis; BP, biological process; GSVA, gene set variation analysis; OR, odds ratio; CI, confidence interval; AUC, area under curve; SVMs, Support Vector Machines; RF, Random Forest; NB, Naïve Bayes; NN, Neural Network; FDA, Flexible Discriminant Analysis; *SIGLEC9*, sialic acid-binding Ig-like lectin 9; *TSPO*, translocator protein; *CKS1B*, cyclin-dependent kinase regulatory subunit 1B; *PTTG3P*, putative pituitary tumor-transforming gene 3 protein; IL, interleukin; SAE, sepsis-associated encephalopathy.
