# Supplementary material for: Integrated Analysis of Gene Co-Expression Network and Prediction Model Indicates Immune-Related Roles of the Identified Biomarkers in Sepsis and Sepsis-Induced Acute Respiratory Distress Syndrome
Source: Front Immunol. 2022 Jun 30;13:897390. doi: 10.3389/fimmu.2022.897390 (PMC9281548; doi:10.3389/fimmu.2022.897390)
Supplement: Supplementary file 1 [file DataSheet_1.docx]

Supplementary Tables

**Table S1. All the identified genes of the key modules associated with sepsis/se-ARDS**

| Key module | Modular genes |
| --- | --- |
| Magenta (263) | *ACAA1, ACAP1, ACPP, ACTN4, ADORA2A, ALDOA, ANPEP, ANXA5, APBB1IP, APCDD1, PLP2, ARF5, ARPC1A, ARPC1B, ARPC4, ATL3, ATOX1, ATXN7L3, BAT5, BCKDK, BLOC1S1, BMX, BRI3, C14orf131, C17orf60, C17orf62, C19orf59, C1orf122, C1orf162, C22orf9, C2orf25, C3AR1, C5orf32, C9orf103, C9orf167, C9orf89, C9orf95, CA4, CACNA1E, CAPNS1, CD14, CD177, CD300LF, CD44, CD63, CD82, CDA, CEACAM3, CEBPA, CKAP4, CNPY3, COG7, COTL1, CST7, CTSA, CTSC, CTSD, CYP1B1, DBNL, DDAH2, DHCR7, DHRS1, DHRS7B, DHRS9, DNM2, EI24, EMILIN2, EMR1, ENO1, EXOC3, EXOSC4, F12, F5, FAM160A2, FAM89A, FBXW2, FCER1G, FERMT3, FES, FIG4, FKBP15, FKBP1A, FLJ22662, FLOT1, FLVCR2, FSTL3, G6PD, GAPDH, GBA, GGT1, GLA, GLT25D1, GM2A, GNA15, GNG5, GPR137B, GPR84, GRB2, GRK6, GRN, GSTO1, GUSB, GYG1, H2AFJ, HAMP, HAVCR2, HCK, HIF1AN, HINT3, HIST1H2BD, HIST1H2BK, HIST2H2AA3, HIST2H2AA4, HIST2H2AC, HIST2H2BE, HK3, HP, IER3, IFI30, IFITM1, IGFBP7, IL17RA, IL1RN, ITGA7, KIAA2013, LAMC1, LDHA, LILRA3, LILRA5, LILRA6, LOC100130520, LOC100130707, LOC100130904, LOC100132717, LOC100133875, LOC255809, LOC391075, LOC440043, LOC440731, LOC606724, LOC642408, LOC642489, LOC644237, LOC644774, LOC644914, LOC648984, LOC651143, LOC653506, LOC653888, LOC723972, LOC728188, LOC728226, LOC728519, LOC728666, LOC729816, LOC730358, LOC731528, LOC732007, LRPAP1, LTA4H, LTB4R, LTBR, METRNL, METTL7B, MMADHC, MMP8, MMP9, MSRA, MSRB2, MTF1, MYO1F, NAPRT1, NAT15, NCKAP1L, NEDD8, NLRC4, NME6, OPLAH, ORM1, OSCAR, OXER1, PADI4, PDSS1, PEX13, PFKFB3, PGAM1, PGD, PGK1, PHCA, PKM2, PLAC8, PLP2, PLSCR1, PNPLA6, POR, PPM1M, PRKAG1, PRMT2, PSMB3, PTGR1, PTK2B, PTPN1, PTPN6, PUS3, PYCARD, RBMS1, RETN, RGL4, RGS19, RNASE2, RPA1, RPS6KA5, S100A12, S100A4, S100A6, S100P, S1PR4, SASH3, SBNO2, SCPEP1, SEC23B, SERPINB1, SERPINB8, SETD8, SGMS2, SGSH, SH2B2, SH3BGRL3, SIGLEC5, SIGLEC9, SIRT7, SLC2A3, SMPDL3A, SP100, SRA1, STX10, STXBP2, SYTL1, TCN2, TDRD9, TIMP1, TKT, TMEM120A, TMEM14B, TNFRSF10D, TOR1A, TPST2, TSPO, TYROBP, UBA1, UBE1, UBTD1, UCK1, UPP1, VAMP3, VAT1, VAV1, VSTM1, VWA5A, WSB1, ZDHHC12, ZDHHC19, ZNF438* |
| Midnight blue (121) | *AFG3L2, ANRIL, ARL2BP, ASPM, ATAD2, ATP1A1, AURKA, AURKB, AZU1, BIRC5, BOLA3, BPI, C13orf34, C16orf75, C3orf26, C6orf173, CAMP, CCNA2, CCNB1, CCNB2, CCNF, CD24, CDC20, CDC45L, CDCA3, CDCA5, CDKN3, CDT1, CEACAM6, CEACAM8, CEBPE, CENPE, CKAP2, CKS1B, CKS2, COL17A1, CPNE3, CTSG, DDIT4, DEFA4, DNAJC9, DTL, ELANE, EPDR1, FBXO5, FEN1, GINS2, HIST1H1C, HMGN2, HMMR, KIF20A, KIF20B, KLF9, KNTC1, LCN2, LDLR, LOC100134379, LOC148137, LOC148915, LOC283392, LOC647000, LOC650412, LOC653061, LOC653600, LOC730534, LTF, LXN, MCM2, MCM6, MCM7, MELK, MGST3, MPO, MS4A3, MYB, NCAPG, NEK2, NNMT, NUSAP1, OIP5, PCCB, PIF1, PIR, PIWIL4, POLE2, PRC1, PRTN3, PTPN20, PTTG1, PTTG3P, QSOX2, RFC4, RNASE3, RRM1, SCD, SGOL2, SLC11A2, SLC27A2, SLC2A5, SPDYE1, SUSD3, TACSTD2, TCN1, TFF3, THBS4, TIMELESS, TIMM23, TK1, TMEM14C, TOP2A, TRIP13, TRMT5, TSC22D3, TUBA1B, TUBA1C, TYMS, UBE2C, UBE2T, UHRF1, WDR51A, ZBTB16* |

**Table S2. Hub genes of the key modules associated with sepsis/se-ARDS**

| Key module | Hub genes |
| --- | --- |
| Magenta (17) | *C19orf59, DDAH2, EXOSC4, FCER1G, FLOT1, GBA, GPR84, GYG1, HK3, LTB4R, OSCAR, PGD, RGL4, SIGLEC9, TDRD9, TSPO, UBTD1* |
| Midnight blue (19) | *AURKA, AURKB, CCNA2, CCNB2, CCNF, CDC20, CDCA5, CDKN3, CKS1B, CKS2, HMMR, NUSAP1, PRC1, PTTG1, PTTG3P, TPX2, TYMS, UBE2C, UBE2T* |

Supplementary Figures


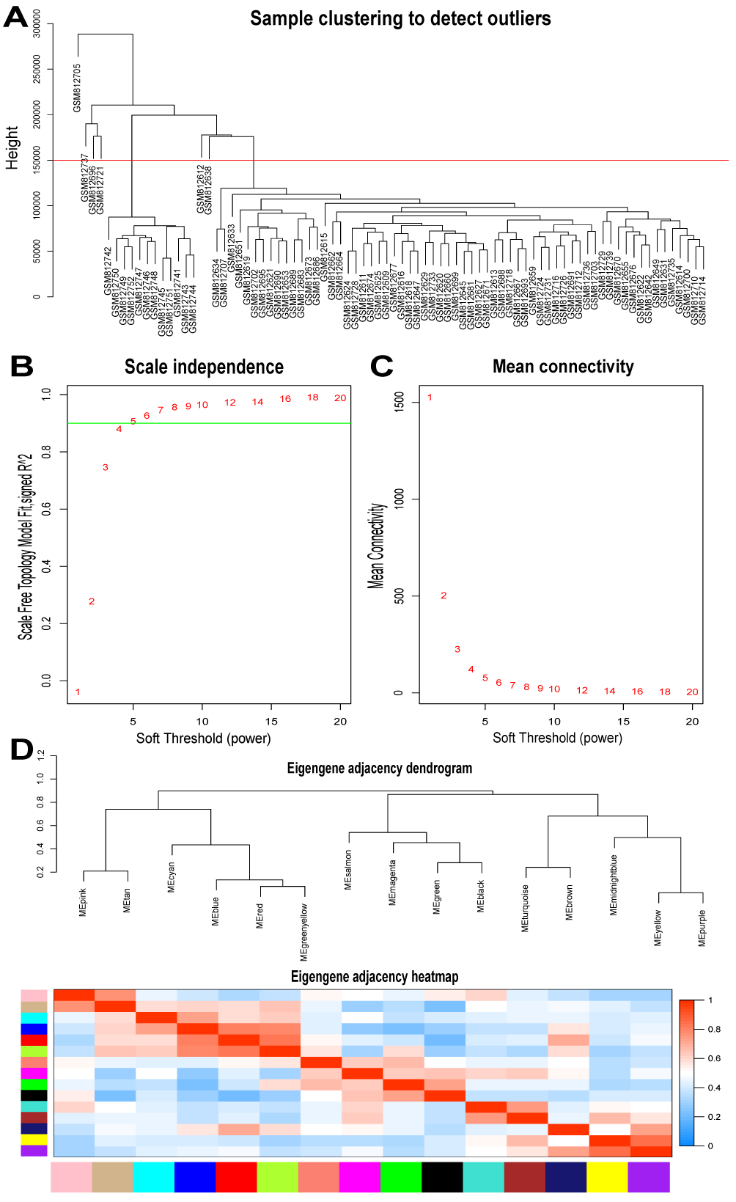


**Figure S1. Construction of weighted gene co-expression networks for sepsis/se-ARDS**

(A) Cluster dendrogram of sepsis/se-ARDS samples with outliers. Scale-free topology model of the scale independence (B) and mean connectivity (C) under different soft-thresholding powers. (D) Hierarchical clustering dendrogram and eigengene network heatmap of the corresponding modules.


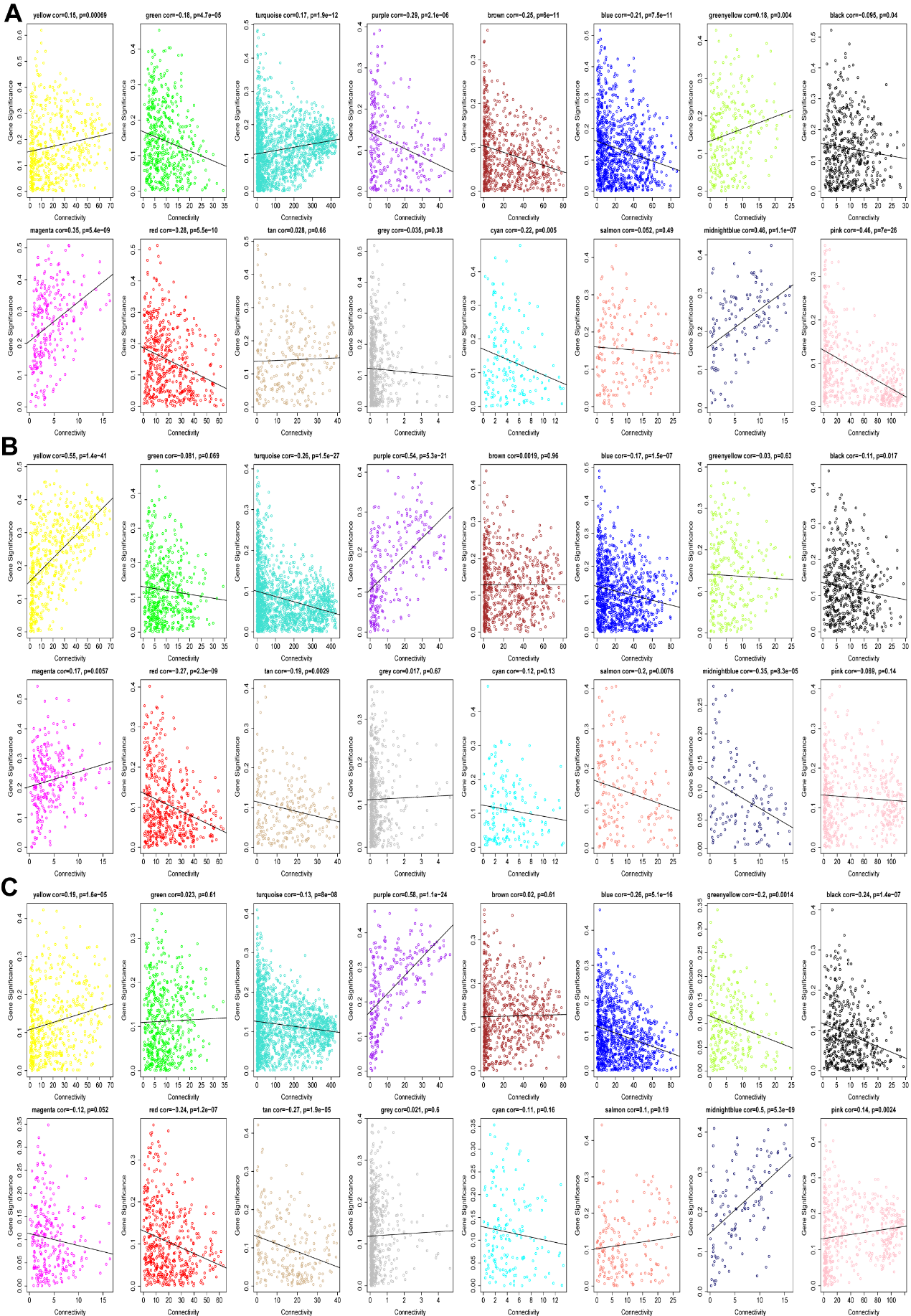


**Figure S2. Analysis of gene significance (GS) and intramodular connectivity of sepsis/se-ARDS**

Relationship between GS and intramodular connectivity of (A) control, (B) sepsis, and (C) se-ARDS groups.


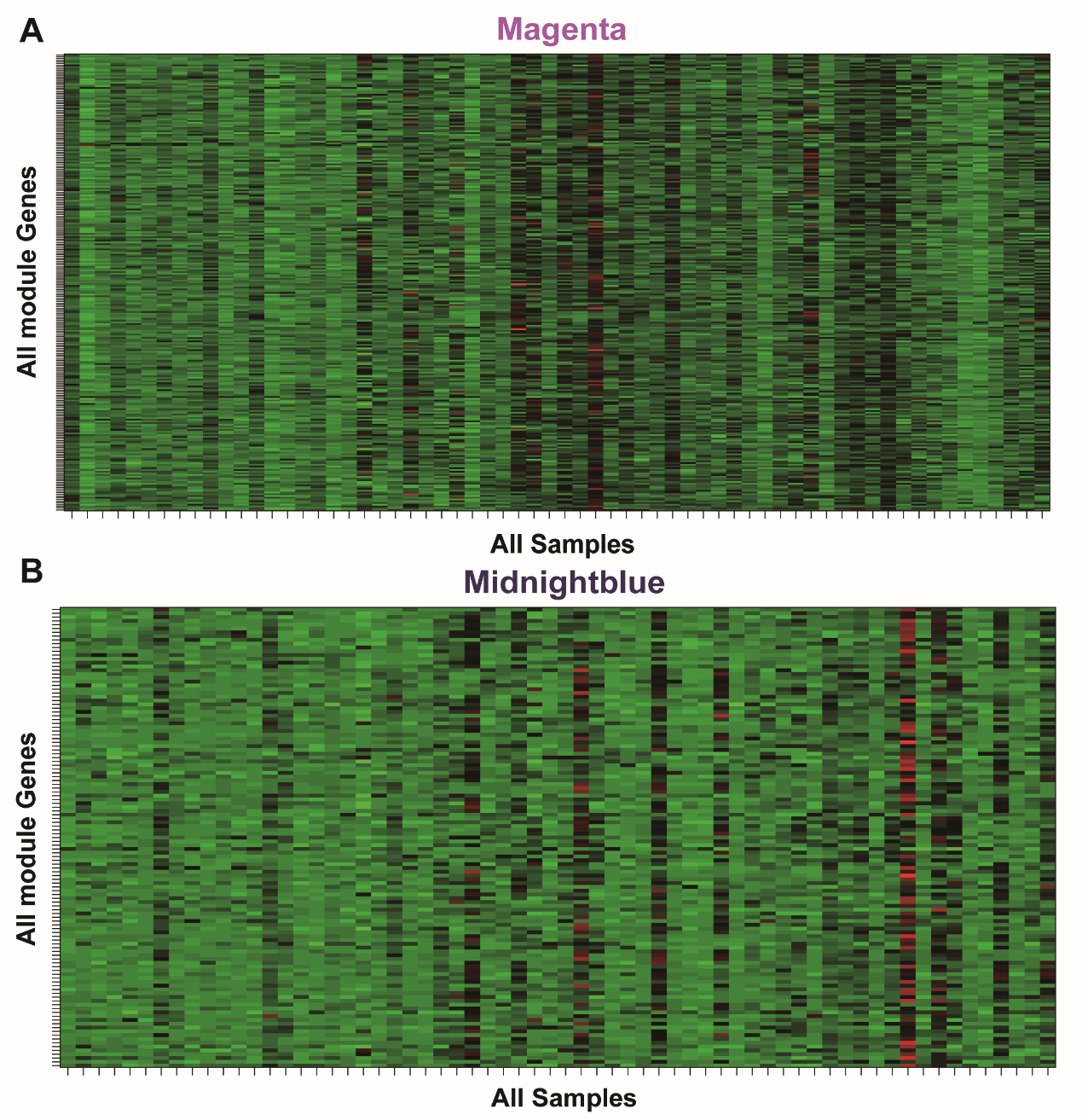


**Figure S3. Expressions of the modular genes for sepsis/se-ARDS**

The heatmaps of the involved genes among all the samples in the identified magenta (A) and midnight blue (B) modules.


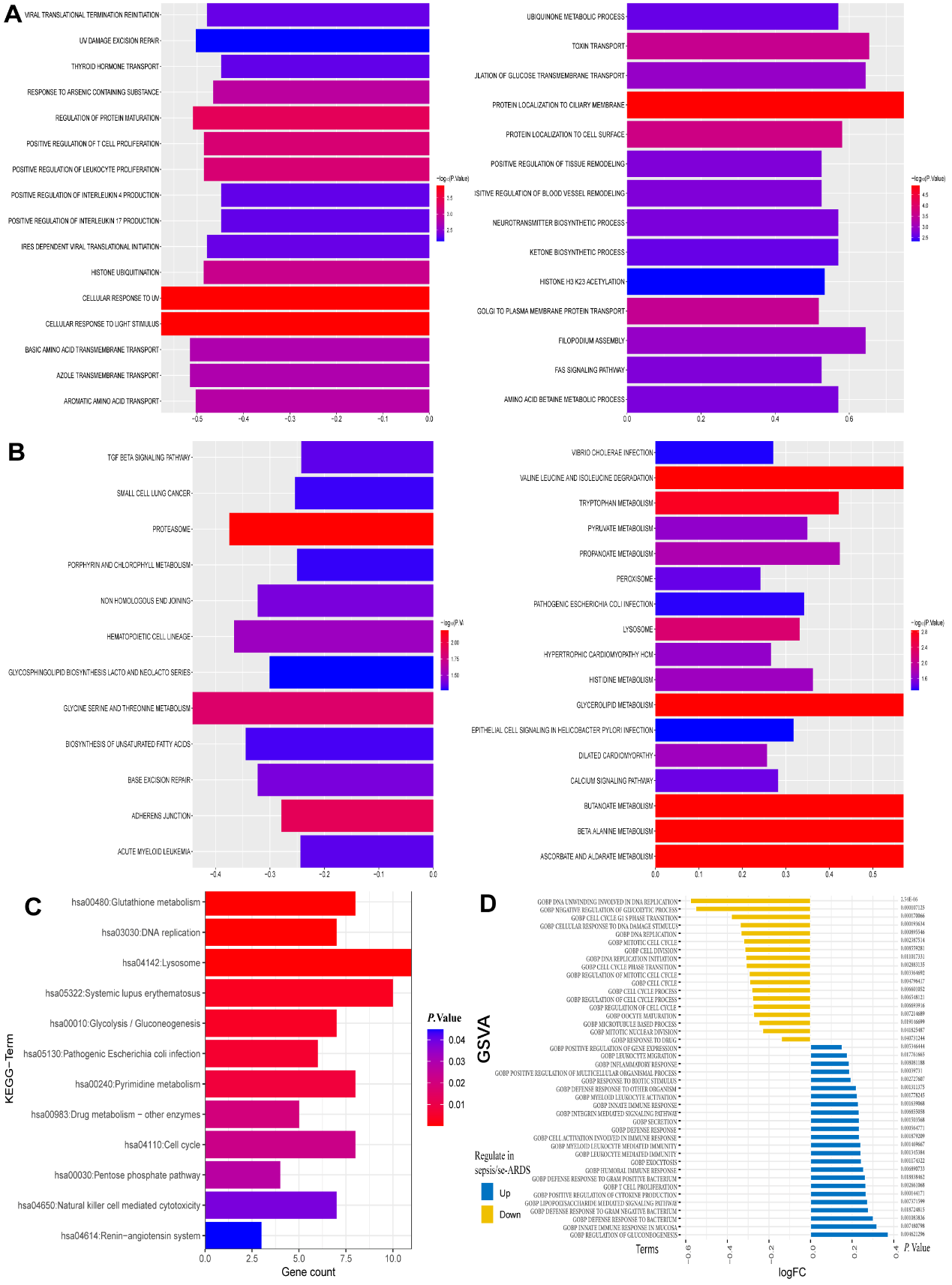


**Figure S4. GO-based biological function and KEGG pathways enriched in sepsis/se-ARDS based on modular genes.**

GSVA-based downregulation (left) and upregulation (right) of (A) GO and (B) KEGG enrichment of the modular genes. (C) KEGG pathway enrichment analysis of DAVID. (D) GSVA-based analysis of biological function enrichment by bar-plot.


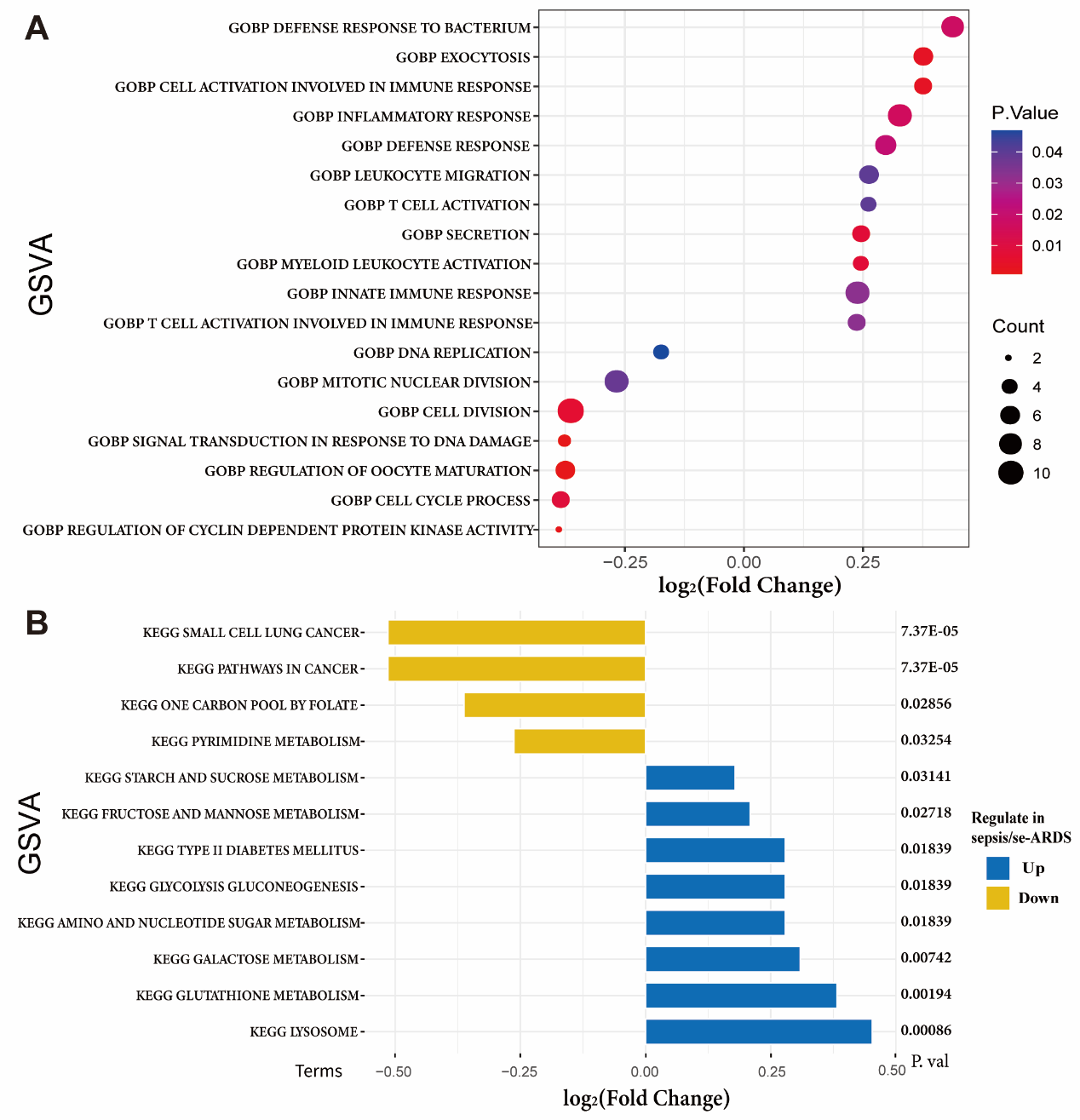


**Figure S5. Functional and pathway enrichment analyses of the hub genes of sepsis/se-ARDS**

(A) GSVA-based analysis of biological function enrichment by bubble plot. (B) GSVA-based analysis of KEGG pathway enrichment by bar-plot.


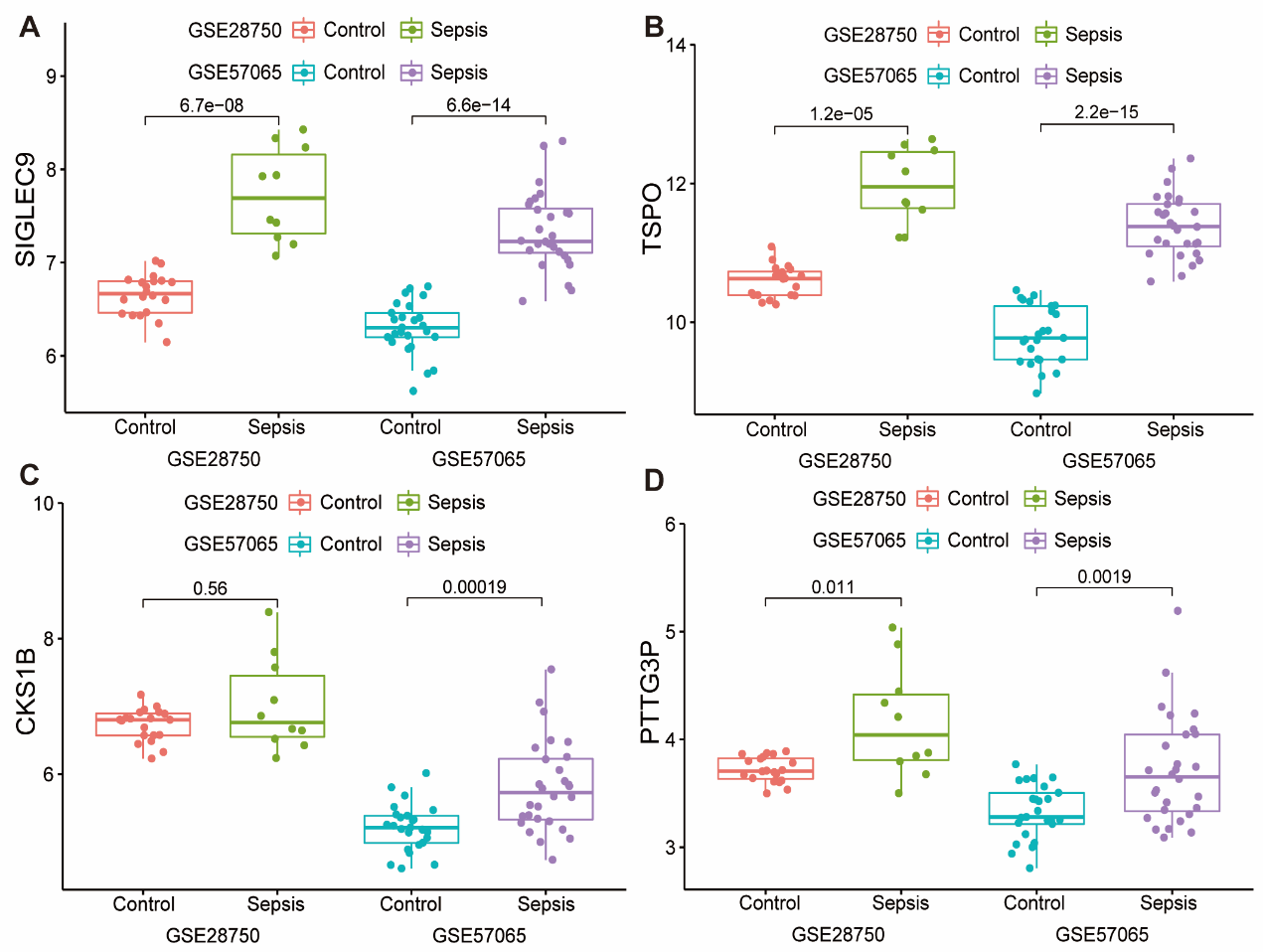


**Figure S6. Expressions of the four marker genes in external cohorts of GSE28750 and GSE57065**

The comparisons of gene expressions of *SIGLEC9* (A), *TSPO* (B), *CKS1B* (C), and *PTTG3P* (D) between groups of control and sepsis patients in GSE28750 and GSE57065.


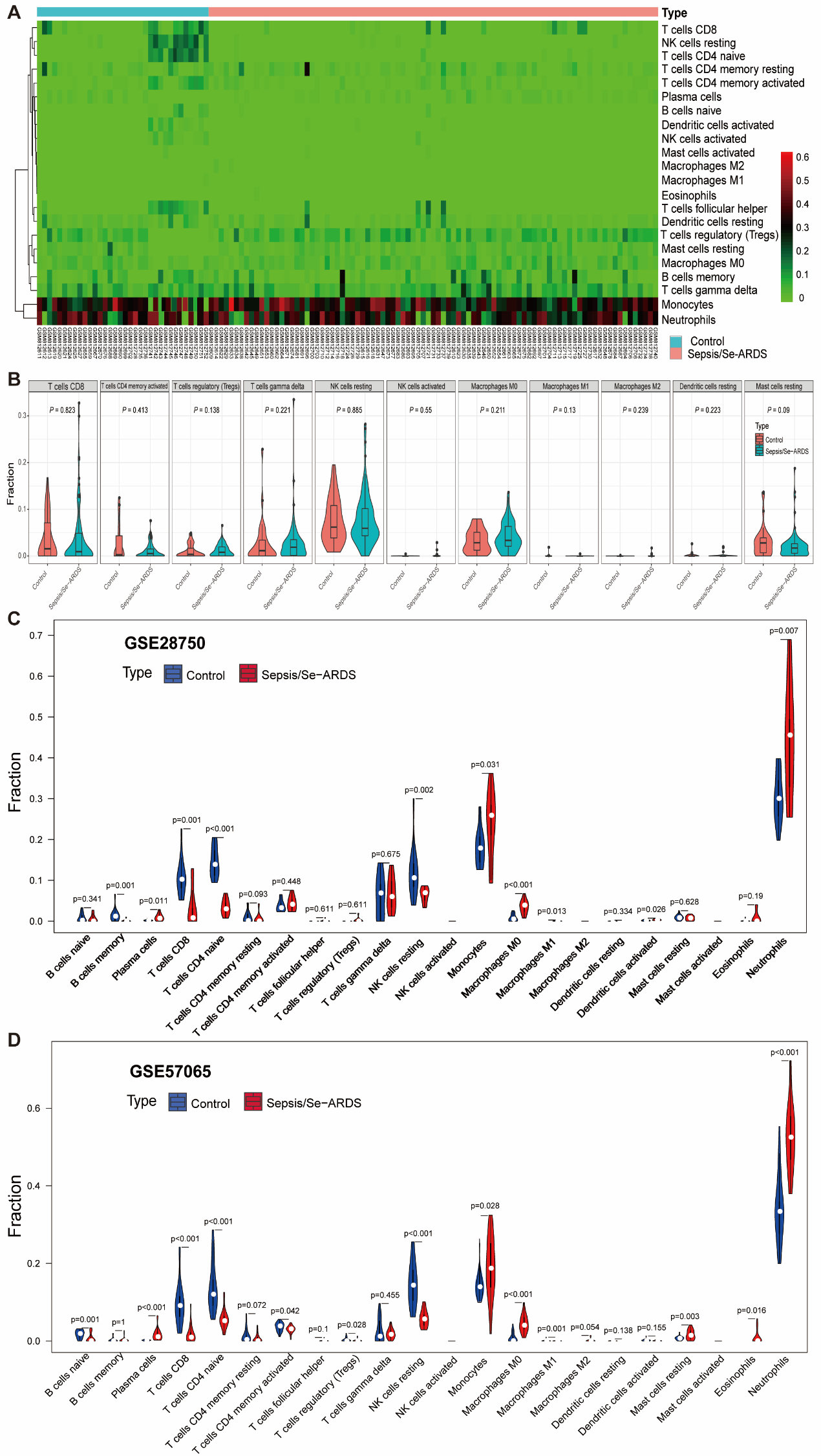


**Figure S7. Profile of infiltrating immune cells**

(A) Relative proportions of the 22 types of infiltrating immune cells among all the samples. (B) Violin plots of the remaining infiltrating immune cells without significant difference between groups in GSE32707. Violin plots of the fractions of 22 types of infiltrating immune cells between control and sepsis groups in GSE28750 (C) and GSE57065 (D).


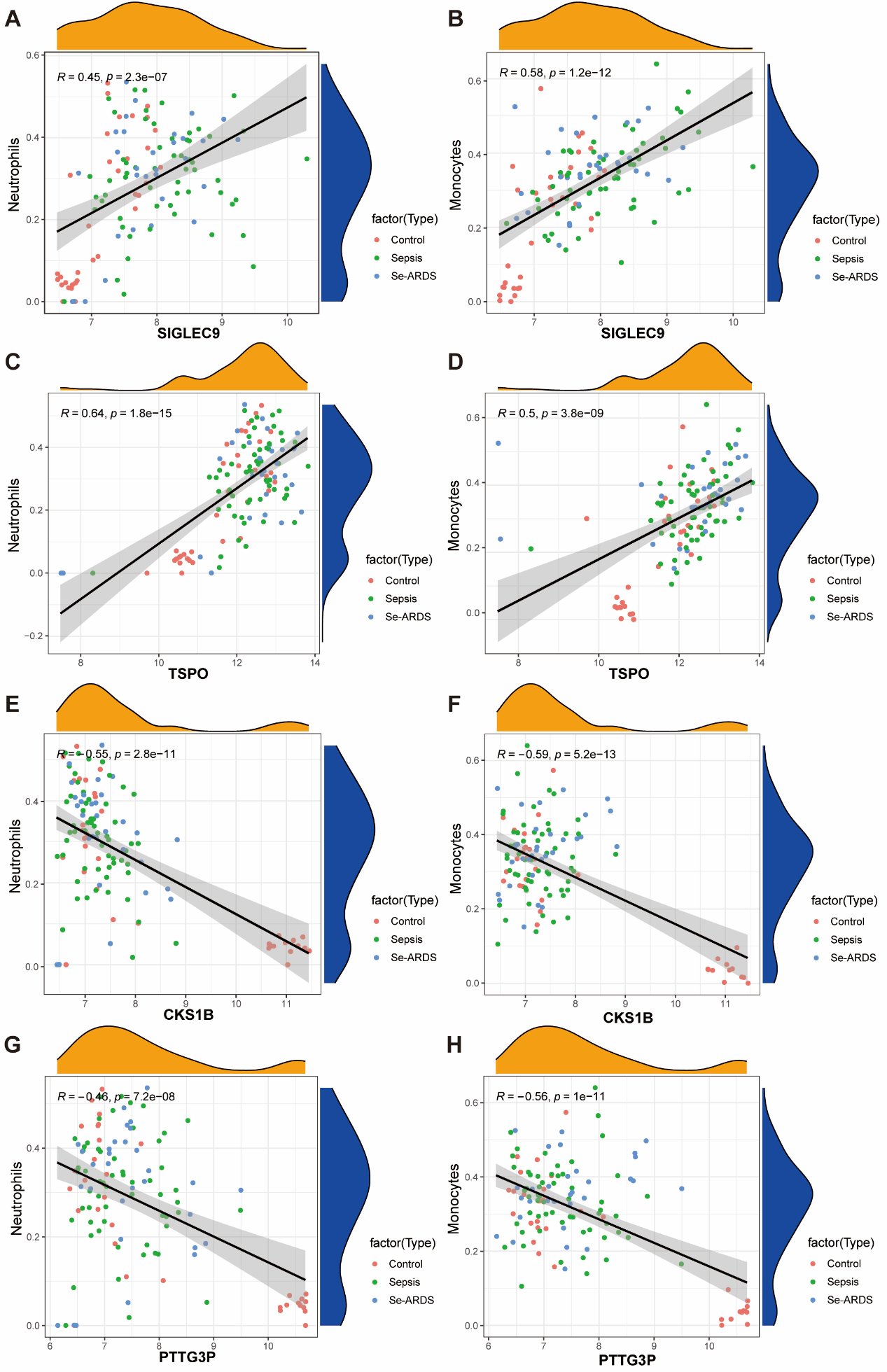


**Figure S8. Correlation of the four biomarkers with the infiltration of specific immune cells**

Correlation analysis of *SIGLEC9* with (A) neutrophil and (B) monocyte infiltration. Correlation analysis of *TSPO* with (C) neutrophil and (D) monocyte infiltration. Correlation analysis of *CKS1B* with (E) neutrophil and (F) monocyte infiltration. Correlation analysis of *PTTG3P* with (G) neutrophil and (H) monocyte infiltration.
